# Microtubule-Mediated Inositol Lipid Signaling Plays Critical Roles in Regulation of Blebbing

**DOI:** 10.1371/journal.pone.0137032

**Published:** 2015-08-28

**Authors:** Tatsuroh Sugiyama, Md. Kamruzzaman Pramanik, Shigehiko Yumura

**Affiliations:** 1 Department of Functional Molecular Biology, Graduate School of Medicine, Yamaguchi University, Yamaguchi, Japan; 2 Microbiology & Industrial Irradiation Division, IFRB, AERE, Bangladesh Atomic Energy Commission, Dhaka, Bangladesh; University of Illinois at Chicago, UNITED STATES

## Abstract

Cells migrate by extending pseudopods such as lamellipodia and blebs. Although the signals leading to lamellipodia extension have been extensively investigated, those for bleb extension remain unclear. Here, we investigated signals for blebbing in *Dictyostelium* cells using a newly developed assay to induce blebbing. When cells were cut into two pieces with a microneedle, the anucleate fragments vigorously extended blebs. This assay enabled us to induce blebbing reproducibly, and analyses of knockout mutants and specific inhibitors identified candidate molecules that regulate blebbing. Blebs were also induced in anucleate fragments of leukocytes, indicating that this assay is generally applicable to animal cells. After cutting, microtubules in the anucleate fragments promptly depolymerized, followed by the extension of blebs. Furthermore, when intact cells were treated with a microtubule inhibitor, they frequently extended blebs. The depolymerization of microtubules induced the delocalization of inositol lipid phosphatidylinositol 3,4,5-trisphosphate from the cell membrane. PI3 kinase-null cells frequently extended blebs, whereas PTEN-null cells extended fewer blebs. From these observations, we propose a model in which microtubules play a critical role in bleb regulation via inositol lipid metabolism.

## Introduction

Various locomotive cells such as neutrophils, fibroblasts, keratocytes, and *Dictyostelium* cells extend lamellipodia via actin polymerization. Actin polymerizes at the leading edge and pushes against the anterior cell membrane, resulting in the extension of lamellipodia [1]. However, certain cells migrate by extending blebs via a process that is independent of the force of actin polymerization [2,3]. Blebs are extended when the cell membrane is locally decoupled and separated from the underlying actin cortex, which induces outward cytoplasmic flow via intracellular pressure. The intracellular pressure (hydrostatic pressure) is generated by the contraction of cortical actin and myosin II [2,4]. The power generated by myosin II appears to be crucial for blebbing, which is mediated by signaling via the small G protein Rho and Rho-associated protein kinase (ROCK) in mammalian cells [3,5].

Bleb-driven migration is especially prominent in three-dimensional environments, such as in collagen gel, whereas lamellipodia predominate during migration on flat surfaces, such as on a coverslip [6,7]. Furthermore, the experimental induction of blebbing enables cells to invade into three-dimensional environments [8,9]. Germ cells move to their correct locations in zebrafish embryos simply by repeated directional blebbing [10]. Some cancer cells can migrate by switching between lamellipodia extension and blebbing, and the extension mechanisms leading lamellipodia and blebs are mutually exclusive [11]. For example, upon knocking down Brick 1, which is a subunit of the WAVE complex that is involved in actin polymerization to drive lamellipodia, HeLa cells extend blebs rather than lamellipodia [12]. A balance between the activities of Rho and Rac is implicated as a signal for the switch [13,14]; however, a comprehensive picture of the signaling scheme for blebbing has not yet been obtained.

Although an abundance of literature exists regarding the physiological role of blebbing, blebs are occasionally considered to be by-products of apoptotic and necrotic processes or as pathological phenomena that occur under physical or chemical stress. However, blebs are not essential for these processes [15] and have recently been recognized as protrusions representing a distinct mode of cell migration. Bleb-mediated cell migration toward chemotactic signals has been reported in fish embryos [10,16] and *Dictyostelium* cells [17].

The cellular slime mold *Dictyostelium* has been studied as a model organism for cell migration, chemotaxis, and cytokinesis [18–22]. *Dictyostelium* cells can extend both lamellipodia and blebs [23]. When these cells are uniformly stimulated with a chemoattractant, they extend blebs [24]. A recent study has revealed that cells extend blebs toward a chemoattractant gradient, indicating that blebs can be integrated into chemotactic cell migration [17]. However, the frequency of bleb extension is too low to be analyzed experimentally in a quantitative manner.

In the present study, we developed a new assay to investigate blebbing in *Dictyostelium* cells. When a *Dictyostelium* cell was cut into two pieces with a microneedle, the anucleate fragment vigorously extended blebs. This assay enabled us to induce blebbing and to identify candidates involved in blebbing regulation in many knockout mutants. After cutting, microtubules in the anucleate fragments immediately depolymerized, followed by bleb extension. The depolymerization of microtubules resulted in delocalization of the inositol lipid PIP3 from the cell membrane. Furthermore, PI3 kinase-null cells extended blebs more frequently, whereas PTEN-null cells extended fewer blebs. From these observations, we proposed a model in which microtubules play a role in blebbing via regulating inositol lipid metabolism.

## Results

### Lamellipodia and blebs in *Dictyostelium* cells

**Fig 1:**
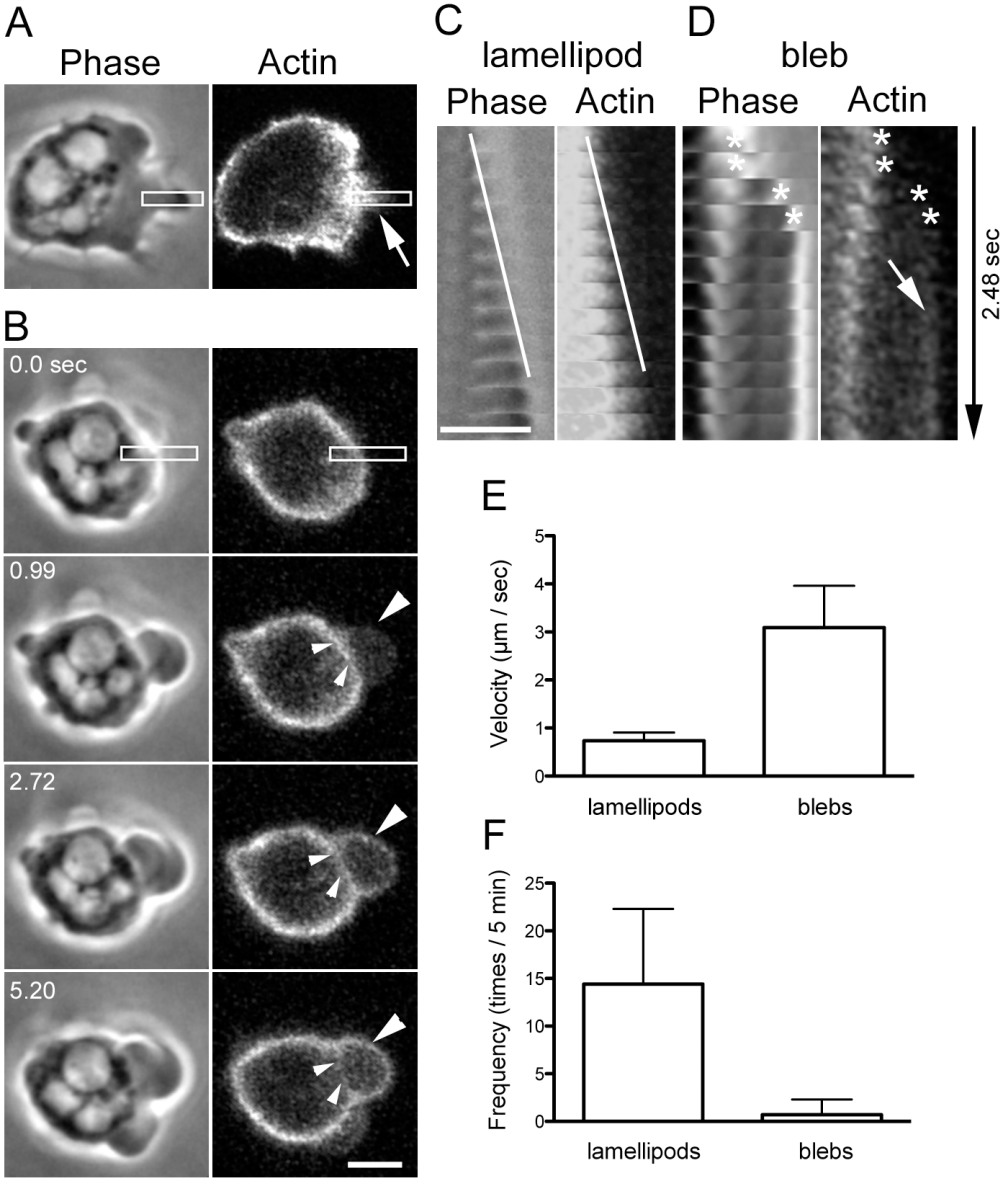
Lamellipodia and blebs in *Dictyostelium* cells. (A and B) Phase contrast and fluorescence images of a typical cell expressing GFP-ABD, an actin filament probe. The cell extended lamellipodia (arrow) followed by blebs (large arrowheads). (C) Kymographs generated from the rectangles in panel A. Note that actin assembly advanced as the lamellipodium extended. Here, the white lines indicate the leading edge of the lamellipodium. (D) Kymographs generated from the rectangles in panel B. A bleb showing a sudden extension (asterisks). Note that actin was not detected along the edge of the initial extension of the bleb and that the actin cortex remained at the basal region of the bleb (small arrowheads in panel B). Approximately 2 seconds later, actin gradually appeared along the edge (arrow). (E) The velocity of lamellipodia and bleb extension (n = 21). (F) The frequency of the appearance of lamellipodia and blebs. Note that lamellipodia were frequently observed (14.4 ± 7.9 times / 5 min), whereas blebs were rarely observed (0.7 ± 1.8 times / 5 min). Bars, 2.5 μm.

*Dictyostelium* cells extend both lamellipodia and blebs. Fig 1A shows live images of a typical cell expressing GFP-ABD, a probe for actin filaments. A representative kymograph shows that actin constantly assembled in the forward direction as the lamellipodia extended (Fig 1C). In contrast, blebs suddenly extended and presented a rounder and smoother shape than lamellipodia (Fig 1B). Actin filaments were not detected along the edges of blebs immediately following extension, and the actin cortex remained at the basal region of the blebs (Fig 1D), suggesting that the cell membrane was detached from the actin cortex and that unlike lamellipodia extension, bleb extension is not driven by actin polymerization. Approximately 1.5 seconds after the blebs extended, actin filaments began to appear along their edges, and the old actin cortex gradually disappeared from the basal region (Fig 1B and 1D). The velocity of bleb extension (Fig 1E, 3.09 ± 0.87 μm / sec, n = 21) was 4 times faster than that of lamellipodia extension (0.73 ± 0.17 μm / sec, n = 21). However, lamellipodia were frequently observed (Fig 1F, 14.4 ± 7.9 times / 5 min, n = 21 cells), whereas blebs were rarely observed (0.7 ± 1.8 times / 5 min, n = 21 cells).

### Induction of blebbing by microsurgery

**Fig 2:**
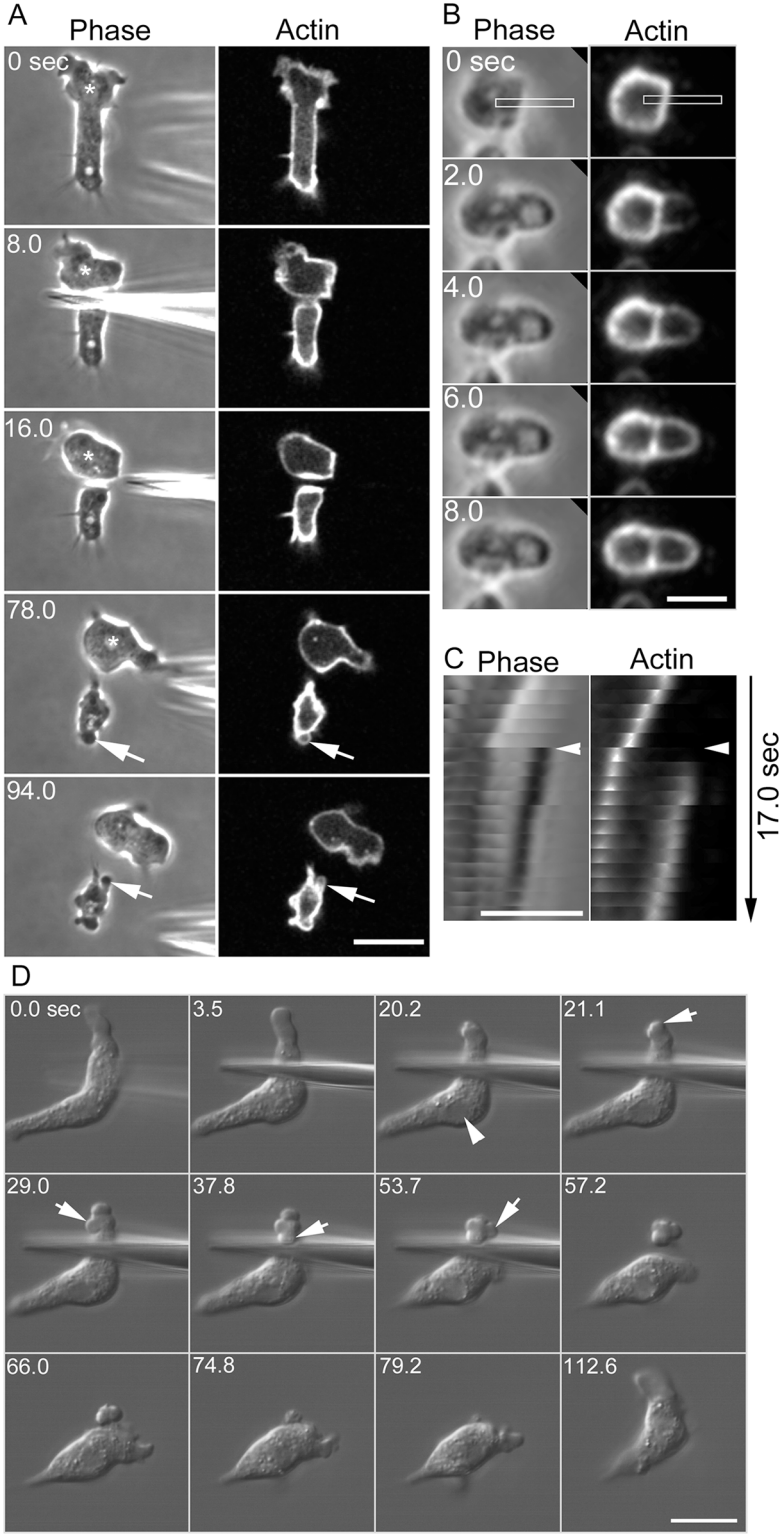
Induction of blebs. (A) A typical microsurgery experiment. A cell expressing GFP-ABD was cut using a microneedle under confocal microscopy. The nucleate fragment continued to migrate, extending a lamellipodium even after cutting. Here, the asterisks indicate the nucleus. The anucleate fragment did not migrate and instead repeatedly extended blebs (arrows). Bar, 10 μm. (B) Time course of bleb-like extension in a typical anucleate fragment. Bar, 2.5 μm. (C) Kymographs generated from the rectangles in panel B. Note that the bleb-like structure extended very quickly and that actin did not localize to its leading edge immediately after extension, suggesting that these extensions from the anucleate fragments are ‘blebs.’ Bar, 2.5 μm. (D) The cytoplasm was disconnected in the middle of the cell by placing a microneedle into the cell under DIC microscopy. After disconnecting the cytoplasm, the half without a nucleus (arrowhead) vigorously extended blebs (arrows), and the other half with a nucleus did not extend them. Both sides of the cytoplasm rejoined when the microneedle was removed, showing that the cytoplasm was not actually cut. After the microneedle was removed, the blebbing ceased. These disconnection experiments could be repeated in the same cells and reversibly induced blebbing. Bar, 10 μm.

Swanson and Taylor [25] showed that when a *Dictyostelium* cell is cut in half, the nucleate fragment resumes normal migration within seconds, while the anucleate fragment does not migrate and instead repeats bleb-like extensions. We first attempted to confirm this observation. Fig 2A shows a typical microsurgery experiment in which a migrating cell expressing GFP-ABD was cut by a microneedle under confocal microscopy. The anucleate fragments frequently extended bleb-like extensions (arrows). Fig 2B shows enlarged sequential images of a typical bleb-like extension of an anucleate fragment. Fig 2C shows a kymograph generated from the rectangles in Fig 2B. The average velocity of their extension (3.33 ± 1.02 μm / sec, n = 24) was approximately comparable to that of bleb extensions in uncut cells. Actin filaments were not detected along the leading edges of the blebs at the initial time of extension, indicating that the extensions in anucleate fragments are ‘blebs,’ as judged from the above criteria. The nucleate fragments did not extend blebs and normally migrated by extending lamellipodia, suggesting that the wound caused by microsurgery does not induce blebbing; rather, the loss of the nucleus or a nucleus-related factor induces blebbing.

### Reversible induction of blebbing

Interestingly, upon disconnecting the cytoplasm in the middle of a cell by placing a microneedle through the cell, the half without a nucleus vigorously extended blebs (Fig 2D), whereas the half with a nucleus did not extend blebs. In this experiment, the cell was not actually cut because both halves of the cytoplasm rejoined, the blebbing ceased, and the cell resumed normal migration once the microneedle was removed. These experiments could be repeated in the same cells to reversibly induce blebbing, supporting the idea that the nucleus or a nucleus-related factor suppresses blebbing.

### Genetic analysis of blebbing

**Fig 3:**
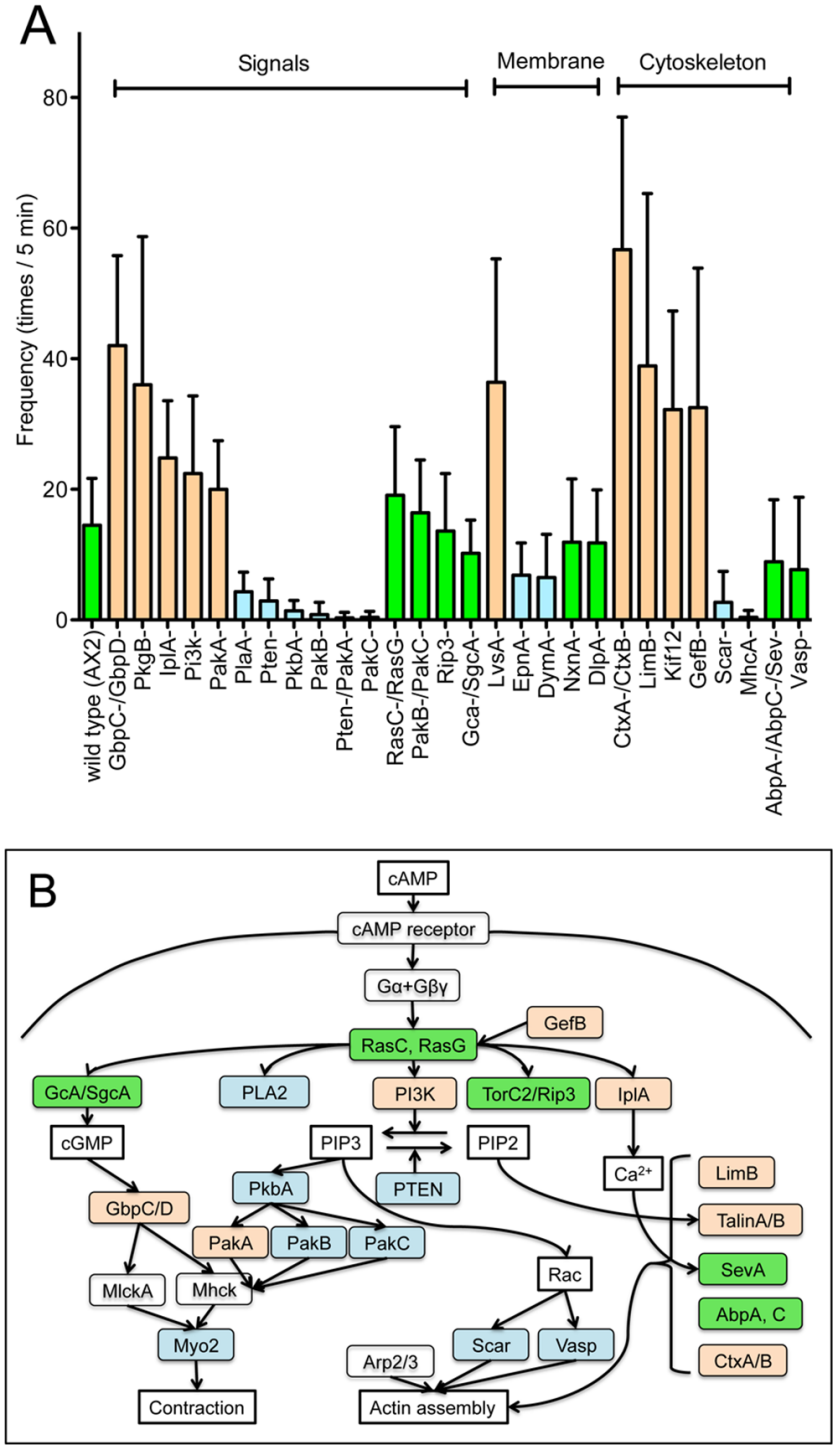
Genetic analysis of blebbing. (A) The frequency of blebbing in various knockout mutants. Knockout mutants in signal proteins, membrane trafficking proteins, and cytoskeleton-related proteins were subjected to the microsurgery-based blebbing assay. Detailed information regarding these mutants is provided in S1 Table. The blebs were counted for 5 min after the mutant cells were cut by microsurgery. (B) Mapping of the assayed genes onto the chemotactic signal network. The binding of cAMP to the cAMP receptor on the membrane activates multiple intracellular signaling cascades, transmits signals to cytoskeletal proteins, and finally drives cell migration toward the chemoattractant. Red, mutants that showed increased blebbing; blue, mutants that showed reduced blebbing; green, no detectable difference compared to wild type cells (as determined by the t-test statics).

There is little information available regarding the intracellular signals that induce bleb formation. Many knockout mutants defective in chemotaxis-, cytoskeleton-, and membrane traffic-related genes are available in the *Dictyostelium* research community. The microsurgery assay enabled us to survey what signals and proteins are necessary for bleb formation. The number of blebs was examined upon cutting mutant cells by microsurgery. Fig 3A summarizes the results (see S1 Table for detailed information regarding the mutants). The mutant cells are categorized into the following three groups: signal proteins, membrane trafficking proteins, and cytoskeleton-related proteins. They extended blebs with varying frequencies. Regarding signal proteins, cells deficient in *gbpC-/gbpD* (guanylate cyclases), *pkgB* (serine/threonine-protein kinase), *iplA* (Ca^2+^ channel), or *pi3k* (phosphatidylinositol 3-kinase) extended blebs more frequently than wild type cells. In contrast, cells deficient in *pkbA* (AKT/PKB), *pten* (phosphatase and tensin homolog), or *pakC* (homolog of mammalian p21-activated kinase) extended blebs infrequently. Regarding membrane trafficking proteins, cells deficient in *LvsA* (contractile vacuole protein) extended blebs more frequently than wild-type cells.

Chemotactic signaling in *Dictyostelium* cells has been extensively investigated; there are 4 main routes of signal transduction from membrane receptor to cytoskeleton [26]. Mutant analysis revealed that the transduction route involving phosphoinositol was clearly involved in the bleb formation, although other routes, such as those involving PLA2 and IplA, may also have roles in this process (Fig 3B). Thus, we focused on phosphoinositol signaling in the latter part of this study.

Regarding cytoskeleton-related proteins, *ctxA/ctxB* (cortexillin)-null cells and *limB* (LIM domain-containing protein)-null cells extended blebs more frequently than wild-type cells, suggesting that these actin-binding proteins contribute to the regulation of bleb formation. In contrast, myosin II-null cells extended virtually no blebs.

### Cortical tension powered by myosin II is required for blebbing

**Fig 4:**
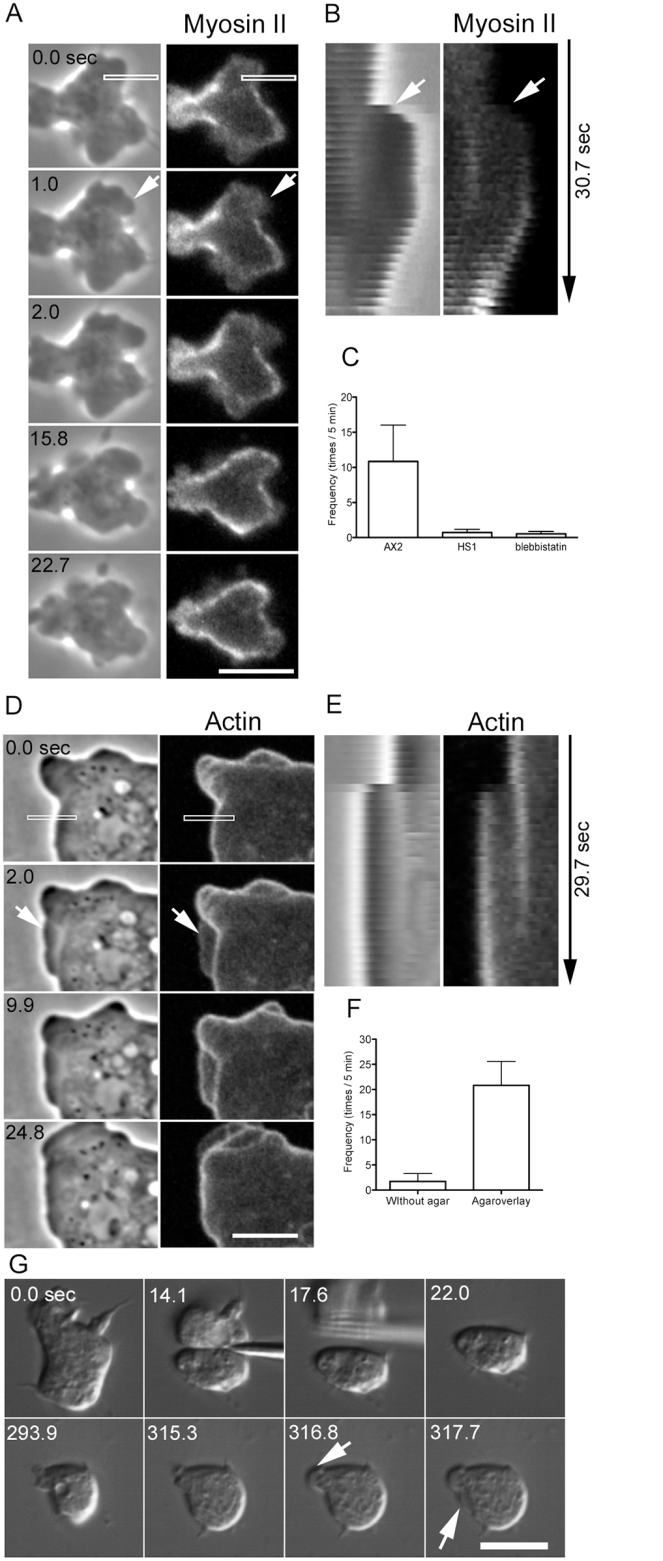
Cortical tension powered by myosin II is required for blebbing. (A) A cell expressing GFP-myosin II was cut, and the dynamics of myosin II in the anucleate fragments were observed under confocal microscopy. Myosin II was not observed along the leading edges of the blebs immediately after extension (arrows), and cortical myosin II remained in the basal region. (B) Kymographs of the dynamics of phase contrast and fluorescence images in the rectangles in panel A. (C) The frequency of blebbing in the anucleate fragments of wild-type (AX2), myosin II-null (HS1), and blebbistatin-treated wild-type cells. (D) A wild-type cell expressing GFP-ABD was observed under pressure applied via an agar block overlay. The cell frequently extended blebs under pressure (arrow). (E) Kymographs of the dynamics of phase contrast and fluorescence images in the rectangles shown in D. (F) Frequency (times per 5-min period) of blebbing with and without an agar overlay (n = 20). (G) After a myosin II-null cell was cut (14.1 sec), the anucleate fragment was pressed with an agar block (293.9–317.7 sec). DIC microscopy shows that the blebs extended under the pressure of the agar (arrows). Bars, 5 μm.

It has been reported that myosin II contributes to bleb formation in various types of cells. We next investigated the role and dynamics of myosin II in the blebbing of cell fragments. Cells expressing GFP-myosin II were cut, and the dynamics of myosin II in the anucleate fragments were observed by confocal microscopy (Fig 4A). In many cases, blebs extended from cortexes that possessed little myosin II. Similar to actin, myosin II was not found along the leading edges of the blebs immediately after extension, and the preexisting cortical myosin II remained in the basal region. Myosin II subsequently appeared along the bleb edge (Fig 4B), followed by bleb retraction, suggesting that the recruitment of myosin II powers the retraction. However, the blebs did not always retract; in some cases, new blebs extended from pre-existing blebs (data not shown).

Next, we investigated whether myosin II contributes to bleb extension. Uncut myosin II-null cells (HS1) did not extend blebs; this observation is consistent with previous works [24]. When myosin II-null cells were cut, virtually no blebs were observed in the anucleate fragments (Fig 4C), although very small and thin extensions were sometimes observed (data not shown). When wild type cells were cut in the presence of blebbistatin, which is an inhibitor of myosin ATPase, virtually no blebs were observed (Fig 4C). Therefore, the activity of myosin II is required for the extension of blebs in anucleate fragments as well.

*Dictyostelium* cells do not have large actomyosin bundles such as stress fibers. Myosin II mainly localizes at the cortex [27,28]. The extension of blebs may be caused by the intracellular pressure generated by the cortical actomyosin. To assess this possibility, pressure was applied to cells expressing GFP-ABD using an agar block. Under this pressure, the cells frequently extended bleb-like extensions (arrows in Fig 4D). Under agar pressure, the morphology of the extensions appeared different from that of blebs not subjected to pressure (Fig 1B). Fig 4E shows a kymograph generated from the rectangle in Fig 4D, indicating that these extensions are ‘blebs,’ according to the criteria mentioned in the previous section. Fig 4F shows the frequency of blebbing in uncut cells with and without pressure, showing that cells under pressure extended blebs more frequently. Myosin II-null cells also extended blebs after being pressed with agar (data not shown). In addition, anucleate fragments in myosin II-null cells also extended blebs upon being pressed with agar (arrows in Fig 4G).

Taken together, these results indicate that myosin II contributes to the extension of blebs by increasing intracellular pressure.

### Microtubule disassembly induces blebbing

**Fig 5:**
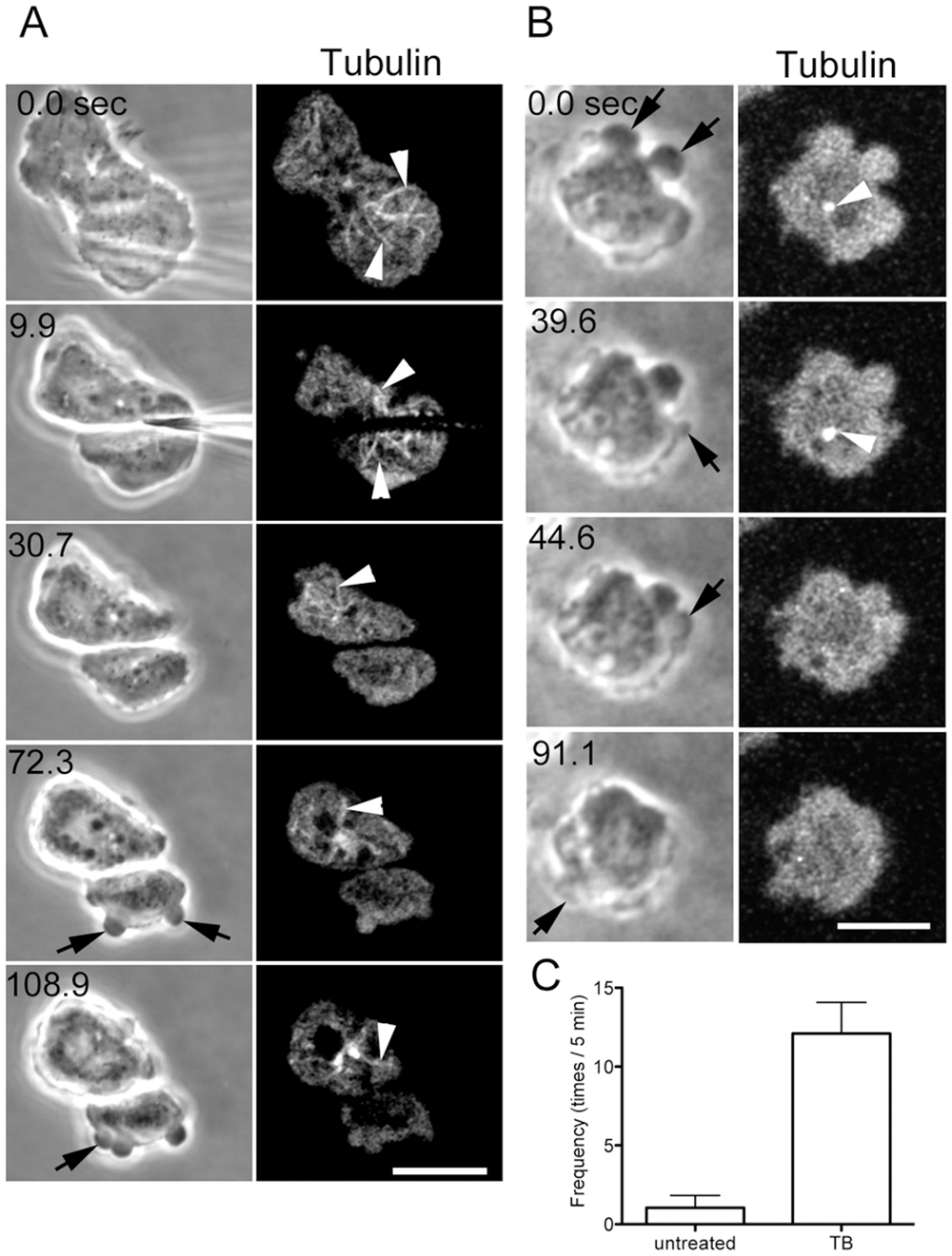
Disassembly of microtubules induced blebbing. (A) When cells expressing GFP-tubulin were cut, the microtubules disassembled within 6.6 ± 4.9 sec (n = 19) in the anucleate fragments, whereas the nucleate fragments retained their microtubule networks (arrowheads). (B) When uncut cells were treated with thiabendazole, most of the microtubules disappeared except at MTOC (arrowheads). These cells frequently extended blebs (arrows). (C) The frequency of blebbing in untreated cells and cells treated with thiabendazole. Bar, 10 μm.

After cells expressing GFP-tubulin were cut, microtubules disassembled in the anucleate fragments, whereas the nucleate fragments retained a microtubule network (Fig 5A). Most likely, the microtubule-organizing center (MTOC), which is firmly associated with the nucleus [29], thereby follows the nucleus to the nucleate fragment after cutting. In the anucleate fragments, the physically cut microtubules must disassemble because they have free minus ends.

Blebbing always began after the microtubules disappeared, suggesting that the disassembly of microtubules induces blebbing (arrows in Fig 5A). To examine this potential role of microtubules, uncut cells were treated with thiabendazole, a depolymerizer of *Dictyostelium* microtubules. In the presence of thiabendazole, a substantial number of microtubules disappeared, except at MTOC (Fig 5B). These cells extended blebs at a high frequency (13.5 ± 3.2 times/5 min; n = 20, Fig 5C).

When the cytoplasm in a cell expressing GFP-tubulin was temporarily disconnected in the middle of the cell by pressing with a microneedle, the microtubules quickly depolymerized in the half that did not have a nucleus. After the microtubules depolymerized, blebbing began in that half (S1 Fig).

Next, cells were cut in the presence of paclitaxel, a stabilizer of microtubules. This drug did not inhibit the blebbing and disassembly of microtubules in the anucleate fragments (data not shown). Paclitaxel was probably ineffective on *Dictyostelium* cell microtubules due to its cell membrane impermeability; indeed, neither cell morphology nor microtubule networks changed in the presence of this drug.

Collectively, these results suggest that microtubules play a role in the suppression of blebbing.

### PIP2 is a key regulator of blebbing

**Fig 6:**
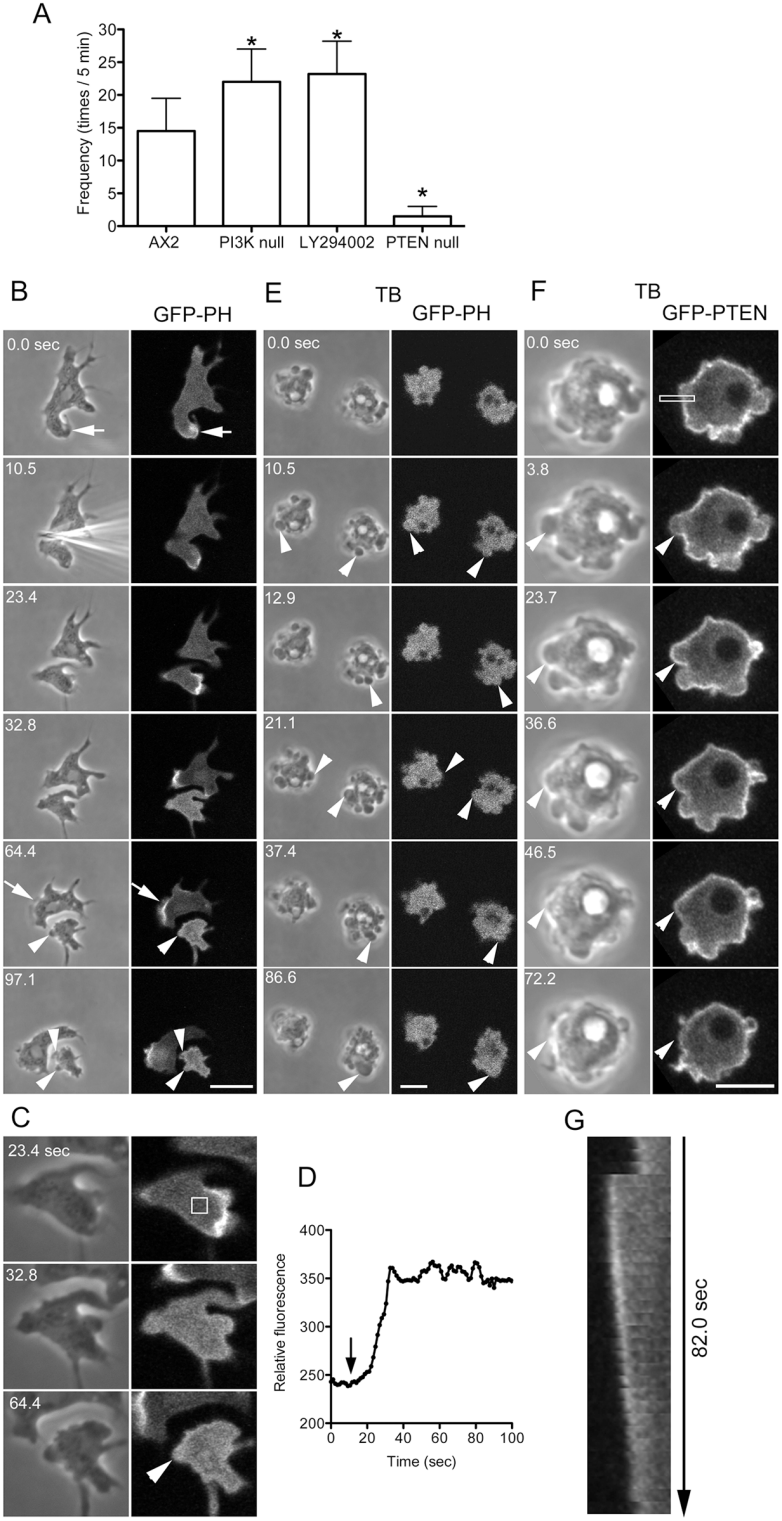
PIP2 is a key regulator of blebbing. (A) Comparison of the blebbing frequencies of anucleate fragments of wild-type, PI3K-null, and PTEN-null cells after cutting. Note that the blebbing frequency was significantly increased in PI3K-null cells and decreased in PTEN-null cells. When wild-type cells were cut in the presence of LY294002, the number of blebs increased (_*_, significant difference compared with AX2, n = 22, p < 0.01). (B) Cells expressing the GFP-PH domain, a marker of PIP3, were cut under confocal microscopy. Time course of phase contrast and fluorescence images before and after cutting. PIP3 was localized along the edges of the lamellipodia (arrow). When the cell was cut into two fragments, the nucleate fragments continued to migrate, and PIP3 was localized along the leading edge (arrow). However, in the anucleate fragment, PIP3 delocalized and became evenly distributed in the cytoplasm. The anucleate fragment frequently extended blebs, but PIP3 did not localize to the blebs (arrowheads). The fluorescence in the blebs appears slightly higher than that in the cytoplasm, which was caused by the exclusion volume [58]. (C) Enlarged images of the fragment shown in panel B. (D) The time course of fluorescence intensity in the fragment (rectangle in panel C), demonstrating that the fluorescence signal increases in the cytoplasm after cutting (arrow). (E) When microtubules were disassembled in cells expressing the GFP-PH domain in the presence of thiabendazole (TB), the cells frequently extended blebs (arrowheads). Note that GFP-PH diffused in the cytoplasm and did not localize to the bleb cortex. (F) Uncut cells expressing GFP-PTEN (G129E), a marker of PIP2, were observed in the presence of thiabendazole (TB). (G) Kymograph of the rectangle in panel F. Note that PIP2 did not localize along the leading edge of the bleb (arrowheads) immediately after extension but rather gradually accumulated there, followed by the retraction of the bleb. Bars, 10 μm.

The intracellular signals for lamellipodia extension have been intensively investigated. In particular, the inositol lipid phosphatidylinositol 3,4,5-trisphosphate (PIP3) plays an important role in actin assembly in lamellipodia [30]. The production of PIP3 is regulated by two enzymes. PI3 kinase (PI3K) phosphorylates phosphatidylinositol 4,5-bisphosphate (PIP2) to generate PIP3. Conversely, PTEN (a phosphatase and tensin homolog) is a phosphatase that dephosphorylates PIP3 to generate PIP2. To investigate the roles of these inositol lipids in blebbing, PI3K-null cells and PTEN-null cells were cut, and the frequency of blebbing was examined. The PI3K-null cells used in this study had mutations in all five PI3K genes. The level of PIP3 in this quintuple PI3K mutant is only approximately 10% of the wild type level [31]. However, the PTEN-null mutant has a higher level of PIP3 than wild-type cells [32]. In the cutting experiments, the anucleate fragments of PI3K-null cells exhibited more frequent blebbing than those of wild type cells, and those of PTEN-null cells showed significantly reduced blebbing (Fig 6A). When wild type cells were cut in the presence of the PI3K inhibitor LY294002, the anucleate fragments frequently extended blebs (Fig 6A).

Next, the localization of PIP3 was observed in live cells expressing the GFP-labeled pleckstrin homology (PH) domain of Akt/PKB, which is an indicator of the location of PIP3. GFP-PH was localized along the leading edge of lamellipodia (arrows in Fig 6B). When a cell was cut into two fragments, the nucleate fragments continued to migrate, and GFP-PH continued to localize along the leading edge. In contrast, in the anucleate fragments, GFP-PH disappeared from the lamellipodia after retraction. Fig 6C shows enlarged images of an anucleate fragment. Fig 6D shows a time course of fluorescence intensity in the cytoplasm (box in Fig 6C), indicating that GFP-PH became evenly diffused throughout the cytoplasm and did not localize to the blebs (arrowheads in Fig 6B and 6C). Incidentally, GFP-PH also did not localize to the blebs in uncut cells (data not shown).

When cells expressing GFP-PH were observed in the presence of thiabendazole, they frequently extended blebs, but GFP-PH did not localize to the blebs (Fig 6E).

We next examined the location of PIP2 in blebbing cells expressing GFP-PTEN (G129E) in the presence of thiabendazole. GFP-PTEN (G129E) is an indicator of PIP2 and has negligible phosphatase activity [33,34]. PTEN was localized along the margins of resting cells and did not localize along the leading edges of blebs immediately after extension; rather, it gradually accumulated there in a similar manner to myosin II (Fig 6F and 6G).

Together, PIP3 localizes to the cortex depending on the presence of microtubules. Most likely, microtubules stabilize PIP3 in the cell membrane, which in turn suppresses bleb formation. PIP2 may positively regulate myosin II in cortex outside of blebs, as discussed later.

### Blebs can be induced in leukocytes by microsurgery

**Fig 7:**
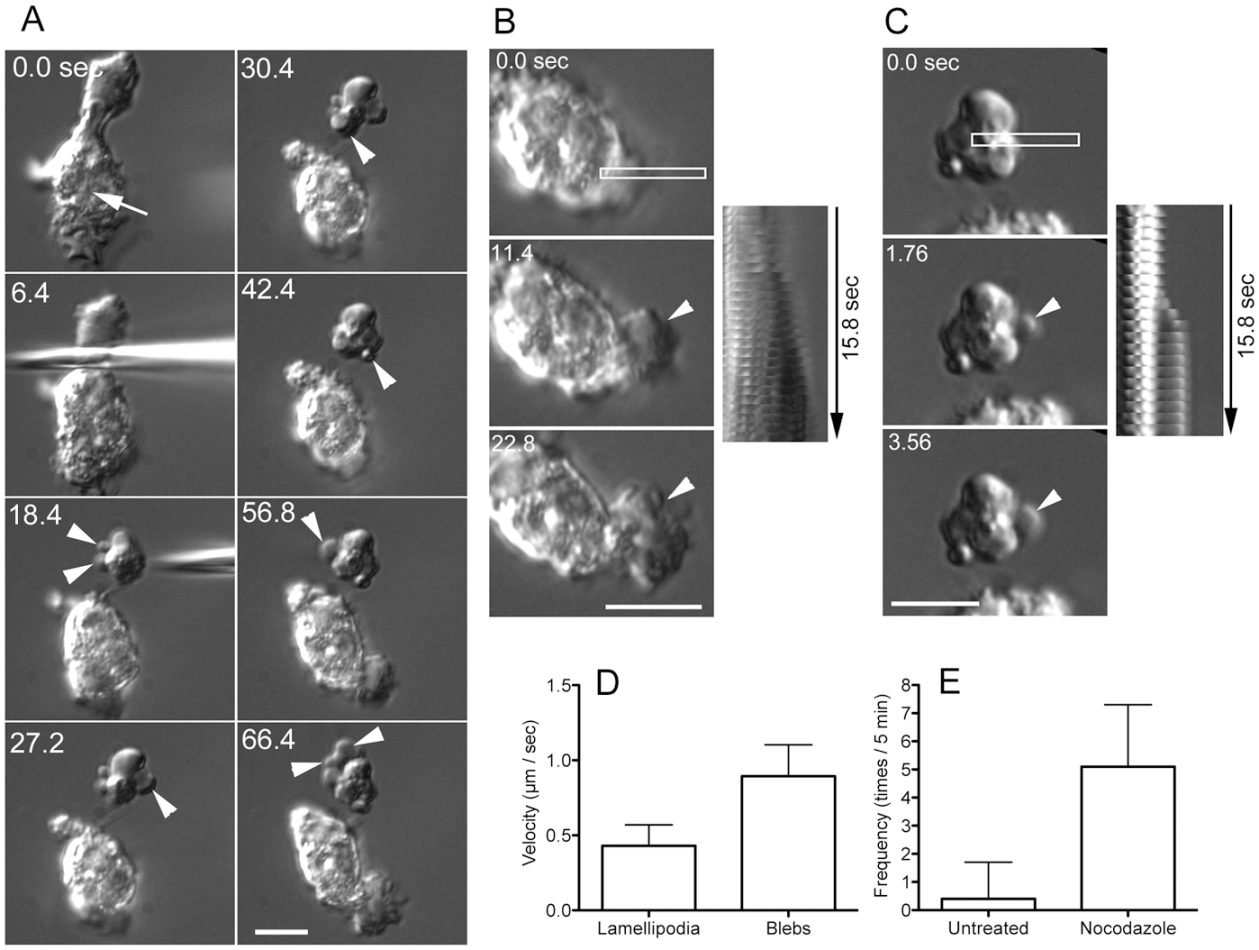
Blebs can be induced in leukocytes by microsurgery. (A) Human leukocytes were individually cut into two fragments under DIC microscopy. The arrow shows the nucleus. After cutting, the nucleate fragment continued normal migration by extending lamellipodia. However, the anucleate fragment frequently extended blebs (arrowheads). (B) Sequential images of lamellipodium extension (arrowhead) and a kymograph generated from the rectangle. (C) Sequential images of bleb extension (arrowhead) and a kymograph generated from the rectangle. (D) Velocities of lamellipodia and blebs. Note that blebs extend much faster than lamellipodia. (E) Bleb frequencies in untreated and nocodazole-treated uncut cells. Note that leukocytes extend more blebs in the presence of nocodazole. Bars, 10 μm.

To investigate whether cutting induces blebbing in mammalian cells, human leukocytes were individually cut into two fragments by microsurgery (Fig 7A). After cutting, the nucleate fragments continued normal migration by extending lamellipodia. Kymograph assays showed that the lamellipodia gradually extended during cell migration (Fig 7B). In contrast, the anucleate fragments frequently and rapidly extended blebs with smooth and spherical morphology (Fig 7C and 7D). When uncut cells were observed in the presence of nocodazole, a microtubule depolymerizer, they extended blebs at a frequency of 4.9 ± 2.5 times/5 min (n = 17), which was significantly higher than that of untreated cells (0.4 ± 1.3 times/5 min, n = 30) (Fig 7E). Therefore, microtubules play an important role in suppressing bleb extension in leukocytes as well as in *Dictyostelium* cells.

## Discussion

In the present study, we developed a new quantitative assay using microsurgery to investigate the signals involved in *Dictyostelium* cell blebbing. Using this assay, blebs could be experimentally induced in a reproducible manner; this was also the case in human leukocytes. The anucleate fragments vigorously extended blebs. The extension of blebs was dependent on the contraction force generated by myosin II because myosin-null cells did not extend blebs. Most likely, the intracellular pressure induced by cortical actomyosin powers the extension of blebs; this pressure could be mimicked by applying pressure to the cells with an agar block. In the anucleate fragments, the microtubules depolymerized after cutting, and the blebs then began to extend. The microtubule inhibitor also induced blebbing in uncut cells, suggesting that the depolymerization of microtubules induces blebbing. The depolymerization of microtubules delocalized the inositol lipid PIP3 from the cell membrane. In addition, PI3K-null cells extended blebs more frequently, whereas PTEN-null cells extended them less frequently than wild-type cells. Therefore, microtubules play a critical role in blebbing-related signaling via inositol lipid metabolism.

Blebs extended by detachment of the cell membrane from the underlying actin cortex. It was previously proposed that this detachment is caused by a local decrease of adhesion between the membrane and the cortex or by local disassembly of the actin cortex [2]. In the present study, no actin cortex disassembly was observed in either the anucleate fragments or in uncut *Dictyostelium* cells, supporting the former scenario.

There are three distinct, successive steps of bleb extension: (1) specification of a bleb site, (2) local detachment of the cell membrane from the actin cortex, and (3) expansion of the bleb. At present, we do not know how the location of bleb extension is specified; microtubules may be the best potential candidate, as is discussed later. Regarding local detachment, a reduction of the attachment energy between the cell membrane and actin cortex must occur locally to initiate blebbing. Indeed, interfering with the activities of linking molecules such as ezrin / radixin / moesin (ERM) and class I myosins frequently induced blebbing in zebrafish prechordal plate mesendoderm progenitor cells [35]. Local pulsed-laser-induced ablation of the cell membrane can induce blebbing [36,37], which also supports the above idea. In the present study, virtually no blebs were observed in the anucleate fragments of myosin II-null cells; instead, very small extensions were often observed. Most likely, blebbing locations are still specified in myosin II-null cells, but without myosin II intracellular pressure is insufficient to induce blebbing, thereby resulting only in small extensions.

Experiments in which membrane tethers were pulled using optical tweezers have shown that decreased PIP2 in fibroblast cell membranes reduces the adhesion between the actin cortex and cell membrane [38]. In *Dictyostelium* cells, live imaging using GFP-PTEN (G129E), an indicator of PIP2, has shown that PIP2 localizes to the rear cortexes in migrating cells and at the furrow regions of dividing cells [33,39,40]. These localization patterns are similar to those of myosin II [27,34]. PTEN-null cells show aberrant myosin II localization, resulting in cytokinesis failure and reduced cell migration. This finding suggests that PTEN is an upstream regulator of proper myosin II localization in cells [34]. In the present study, both PTEN and myosin II mainly localized along the cortex, except in blebs of the anucleate fragments. The observation that blebs extend at cortical regions that possess less PIP2 is consistent with the results of the membrane tethering experiments, which indicated that PIP2 increases the strength of adhesion between the actin cortex and cell membrane [38]. Recent observations of blebs in *Xenopus* primordial germ cells and of fixation-induced blebs in human leukemia cells also indicate the important role of PIP2 in bleb formation [41,42].

Furthermore, because PIP2 localizes to the cortex rather than to bleb sites, it can induce the localization of myosin II to the same region and enhance actomyosin contraction, resulting in increased intracellular pressure and bleb expansion.

Some cells, such as Walker 256 carcinosarcoma cells and *Dictyostelium* cells, can switch between lamellipodia-driven and blebs-driven modes [11,24]. If lamellipodia are prominent in a cell, blebs are not frequent in that cell. This inverse correlation suggests that lamellipodia and blebs are regulated in a reverse manner via common upstream regulators. PIP3 is localized to the leading edges of migrating *Dictyostelium* cells and induces actin polymerization to extend lamellipodia [43]. Therefore, as shown in the present study, the amount of PIP3 in a cell cortex is a strong candidate regulator of this switching. Switching regulators in other organisms have been reported. In the course of zebrafish gastrulation, noncanonical Wnt signaling regulates the balance between lamellipodia-driven (mesenchymal mode) and blebs-driven cell migration by regulating myosin activity via the Rho pathway [44,45]. In melanoma tumor cells, a balance between the activities of the small GTPases Rac and Rho is important in switching between lamellipodia-driven and blebs-driven cell migration [14]. Inhibiting activities of either of these small GTPases has been shown to induce switching between the modes of migration [13,14,46].

In the present study, we demonstrated that microtubules stabilize PIP3 in the cell membrane, likely by attaching to the cell cortex via their distal ends. Although the molecular mechanism for PIP3 stabilization is not clear at present, some signals are probably carried along microtubules to activate PI3K or inactivate PTEN at the cell cortex. It has been reported that microtubules play an important role in the polarization of cells for chemotactic migration and proper cell division. Tsunami, a Hedgehog signaling protein kinase, localizes to microtubule networks in *Dictyostelium* cells and is related to PIP3 production and actin assembly. Mutant cells deficient in *tsunami* show a defect in chemotaxis due to their inability to become polarized and to correctly orient lamellipodia in chemoattractant gradients [47]. The 14-3-3 protein coordinates microtubules and the actin cytoskeleton to control cell mechanics and cytokinesis [48]. *Dictyostelium* Lis1, originally identified as a target for the sporadic mutations that cause lissencephaly in humans, localizes to the microtubule network and modulates actin dynamics by binding to Rac1A [49]. EB1, a microtubule end-binding protein, is required for the intracellular localization of SCAR/WAVE, an actin nucleation factor [50].

**Fig 8:**
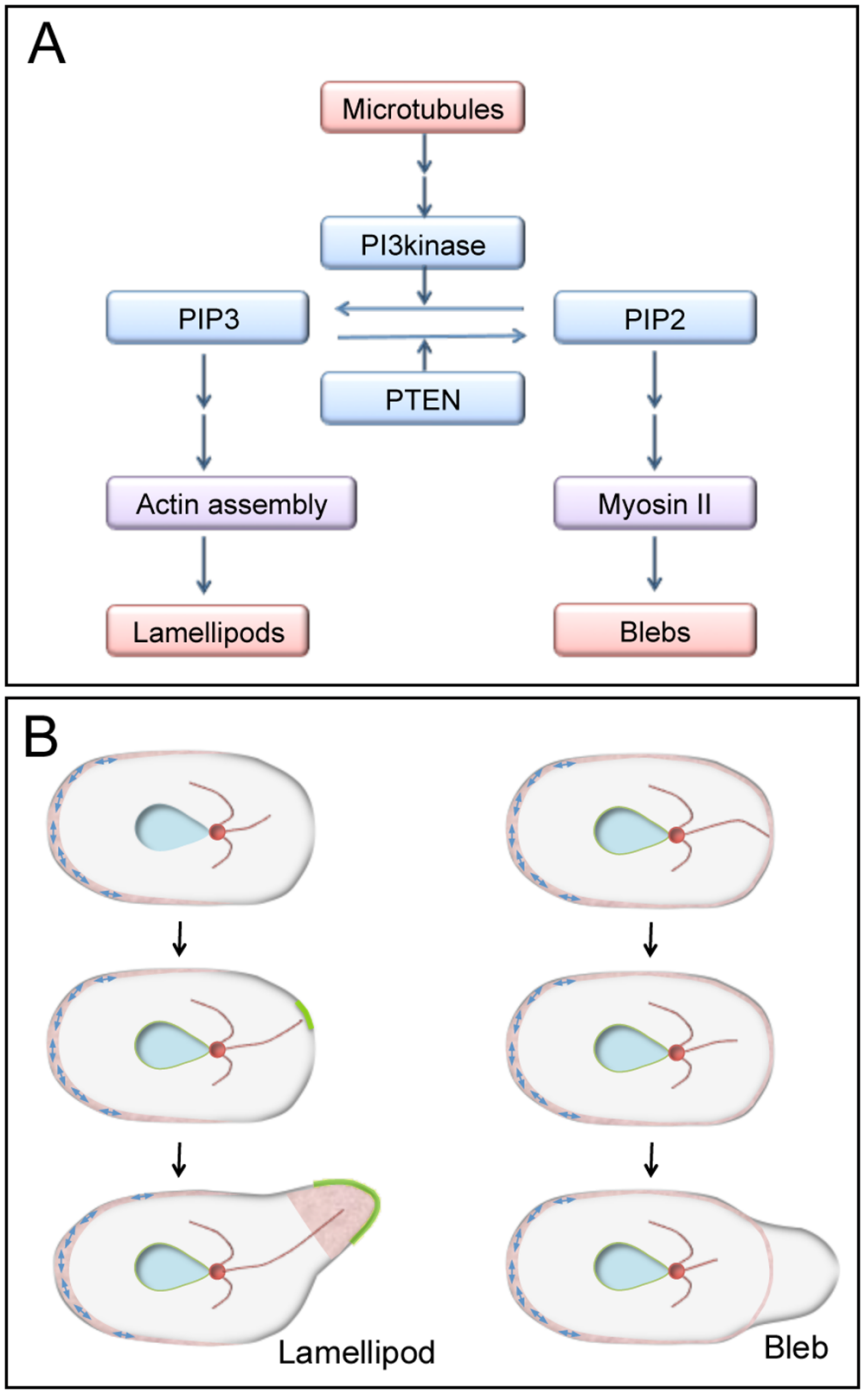
Current model for blebbing signaling. (A) The results of our present study indicate that microtubules can be positioned upstream of bleb extension. In the presence of microtubules, PIP3 localizes to the anterior membrane and induces lamellipodia formation by enhancing the assembly of actin at that location. Without microtubules, PIP3 delocalizes from the cell membrane. PIP2 recruits myosin II along the cortex, generating more internal pressure inside of the cell. A bleb will extend at the weakest part of the cortex (where the levels of PIP2 and myosin II proteins are low) because myosin II contributes to the rigidity of the cortex. (B) A model of lamellipodium and bleb extension in uncut cells. When microtubules extend to the cortex, unknown signals are conveyed along the microtubules that locally induce PIP3 accumulation and actin assembly in the cortex, resulting in the extension of lamellipodium (left). However, when microtubules retract or detach from the cortex, PIP3 delocalizes from the cortex. In the presence of intracellular pressure, based on the power of myosin II, the cell membrane detaches from the weakest part of the actin cortex, resulting in bleb formation. Blue double arrows, myosin II; red, actin filaments; green, PIP3.

Taken together, our data lead us to propose a model in which microtubules play a critical role in bleb extension-related signaling via inositol lipid metabolism (Fig 8A). In uncut cells, when microtubules detach from the cell cortex by physical displacement or via shortening due to dynamic instability, PIP3 locally delocalizes from the cell cortex. This delocalization occurs due to PI3K inactivation or PTEN activation, which may specify the location of blebbing (right in Fig 8B). PIP2 localizes to the cortex, except in nascent blebs, and recruits and activates myosin II, resulting in bleb expansion by increasing the intracellular pressure. However, when microtubules attach to the cortex, PIP3 is locally enriched in the cell membrane, resulting in polymerization of actin filaments and extension of lamellipodia (Fig 8B, left). At present, there is no direct evidence of such a role for microtubules in migrating *Dictyostelium* cells, but it is known that astral microtubules can induce actin polymerization that results in development of mitosis-specific dynamic actin structures (MiDAS), a substratum adhesion, in dividing *Dictyostelium* cells [51]. It is also known that dynamics of microtubules affects blebbing during fibroblast spreading [52]. In epithelial cells, the depolymerization of microtubules by colcemid activates RhoA/ROCK, thus leading to blebbing [53].

Blebs are prominent in cells in 3D environments, such as cancer cells and primordial germ cells. In such constricted environments, cells receive outside pressure, which increases their intracellular hydrostatic pressure, and may stimulate bleb extension, as shown by agar overlay experiments in the present study. Alternatively, the pressure of the agar overlay may separate the distal ends of the microtubules from the cortex, thereby inducing blebbing.

After the blebs extend, they retract into the cell body in many cases. Before the blebs retract, actin and myosin II accumulate along the underlying cortex of the blebs, and the retraction ends within 10–30 sec. A series of actin and myosin II relocation events must occur; these events are spatiotemporally regulated during bleb extension and retraction. The mechanism by which actin and myosin II accumulate along the cortex with appropriate timing remains to be clarified; this remains an unsolved question that is fundamental to our understanding of the molecular mechanisms of cell migration and cytokinesis.

In conclusion, our findings demonstrate that microtubules play a critical role in regulating blebbing via inositol lipid metabolism. Many intracellular signals, actin-related proteins, and membrane trafficking proteins participate in blebbing. The complete picture of the signaling cascade, from microtubule changes to bleb extension, remains to be clarified in the future.

## Materials and Methods

### Cell Preparation

*Dictyostelium discoideum* wild type (AX2) cells and mutant cells were cultured in plastic dishes at 22°C in HL5 medium (1.3% bacteriological peptone, 0.75% yeast extract, 85.5 mM D-glucose, 3.5 mM Na_2_HPO_4_ ·12H_2_O, and 3.5 mM KH_2_PO_4_, pH 6.3). The sources of these cells are listed in S1 Table. GFP-ABD, GFP-myosin II, GFP-∂-tubulin, and GFP-PH domain expression vectors were transformed by electroporation, as previously described [54]. Transformed cells were selected in HL5 medium supplemented with 10 μg/ml G418 (Sigma). The medium was exchanged with BSS (3 mM CaCl_2_, 10 mM KCl, 10 mM NaCl, and 3 mM MES, pH 6.3) and cells were incubated in the solution for 6–8 h. Details regarding the mutant strains are presented in S1 Table.

Neutrophils were isolated using an established method [55]. Briefly, blood was sampled by finger pricking with Medisafe Finetouch (Terumo). The authors were the source of blood. Approximately 50 μl of blood was placed in a glass-bottom chamber at 37°C. After 45 min, the clot was removed and washed repeatedly with HBS (1 mM MgCl_2_, 1 mM CaCl_2_, 5 mM KCl, 150 mM NaCl, and 20 mM HEPES, pH 7.3) supplemented with 10 mM D-glucose, 0.2% (w/v) bovine serum albumin (BSA), and 20 nM formyl-Met-Leu-Phe.

### Microscopy

Cells were placed in a glass-bottomed chamber and observed under a differential interference contrast (DIC) or phase contrast microscope (IX71, Olympus). Cells expressing GFP-ABD, GFP-myosin, GFP-tubulin, or GFP-PH were observed under a confocal microscope (LSM 510 META, Carl Zeiss) equipped with a 100x objective lens (Plan-Neofluar NA x 1.3) as previously described [56]. A 488-nm argon laser beam was used for excitation of GFP, and fluorescence was detected using a 505-530-nm band-pass filter.

### Microsurgery

Glass microneedles were made from glass rods (GD-1.5, Narishige) using a puller (PG-1, Narishige). Each was bent at a 25-degree angle with a micro-forge (MF-830, Narishige). The tips of the microneedles were dipped in Sigmacote (Sigma) to make them hydrophobic, thereby preventing the cells from sticking to the microneedle. The microneedle was mounted on a manipulator (3Man, S Company) that was attached to an inverted DIC microscope or a confocal microscope. To cut the cells, the microneedle was placed onto a cell and quickly pulled.

For the cell pressing experiments, an agar block (0.17-mm thick, 1.5% agarose) was prepared as described previously [27]. The cells were cut using a microneedle under the agar block, and the external solution was then carefully removed with a piece of filter paper to apply pressure to the cells or cell fragments.

### Inhibitors

Blebbistatin, thiabendazole, nocodazole, and LY294002 were dissolved in dimethyl sulfoxide (DMSO) to create stock solutions of 20 mM, 100 mM, 10 mM, and 5 mM, respectively. The final concentrations of these inhibitors for the experiments were 150 μM, 0.1 mM, 17 μM, and 20 μM in BSS. The experiments with blebbistatin, nocodazole and LY294002 were conducted after the cells were incubated in the presence of each inhibitor for 0.5–1 h. As a control, DMSO (0.25%) alone was added, which did not affect either cell morphology or blebbing either before or after cutting.

To completely depolymerize their microtubules, *Dictyostelium* cells were incubated on ice for 30 min in the presence of thiabendazole [57]. These cells were observed by confocal microscopy in the presence of thiabendazole at 22°C. Under these conditions, all of the microtubules except at the microtubule organizing center (MTOC) were completely depolymerized.

Nocodazole (17 μM) in HBS (supplemented with D-glucose plus BSA and 20 nM formyl-Met-Leu-Phe) was used to depolymerize the microtubules in neutrophils.

### Image analysis

Time courses of the extension and retraction of lamellipodia and blebs were analyzed by creating kymographs in Image J (http://rsbweb.nih.gov/ij/).

### Ethics

This study was conducted in accordance with the ethical standards of the Helsinki Declaration.

## Supporting Information

S1 Fig Microtubules reversibly depolymerize in anucleate halves of cells in disconnection experiments. When the cytoplasm of a cell expressing GFP-tubulin was disconnected by pressing with a microneedle under confocal microscopy, the microtubules quickly depolymerized in the anucleate half. After the microtubules depolymerized, blebbing began (arrows). Both sides of the cytoplasm rejoined after removing the microneedle, and the blebbing then ceased. After joining, the cell again had an intact network of microtubules. Bars, 10 μm.
(TIF)

S1 Table Blebs in mutant cells. The frequency of blebs was examined in mutant cells for 5 min after cutting. The frequency is plotted in the graph in Fig 4A. Most of the mutants were provided by the DictyBase stock center (www.Dictybase.org). ‘n’ represents the number of examined cells.
(DOCX)
