# Supplementary figures and images for: Microtubule-Mediated Inositol Lipid Signaling Plays Critical Roles in Regulation of Blebbing
Source: PLoS One. 2015 Aug 28;10(8):e0137032. doi: 10.1371/journal.pone.0137032 (PMC4552846; doi:10.1371/journal.pone.0137032)

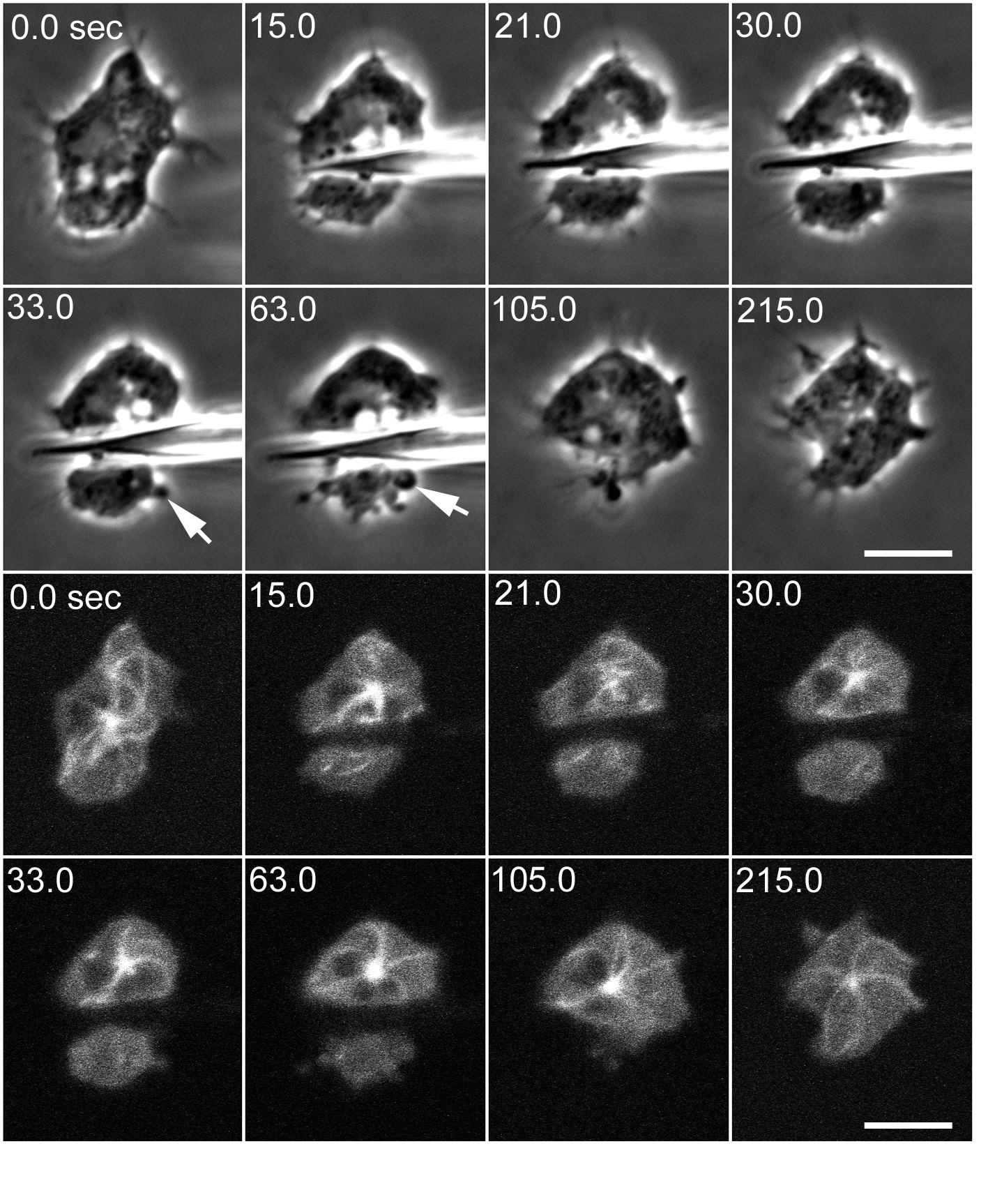

Supplement: S1 Fig — When the cytoplasm of a cell expressing GFP-tubulin was disconnected by pressing with a microneedle under confocal microscopy, the microtubules quickly depolymerized in the anucleate half. After the microtubules depolymerized, blebbing began (arrows). Both sides of the cytoplasm rejoined after removing the microneedle, and the blebbing then ceased. After joining, the cell again had an intact network of microtubules. Bars, 10 μm. (TIF) [file pone.0137032.s001.tif]
