# Supplementary material for: Microtubule-Mediated Inositol Lipid Signaling Plays Critical Roles in Regulation of Blebbing
Source: PLoS One. 2015 Aug 28;10(8):e0137032. doi: 10.1371/journal.pone.0137032 (PMC4552846; doi:10.1371/journal.pone.0137032)
Supplement: S1 Table — The frequency of blebs was examined in mutant cells for 5 min after cutting. The frequency is plotted in the graph in Fig 4A. Most of the mutants were provided by the DictyBase stock center (www.Dictybase.org). ‘n’ represents the number of examined cells. (DOCX) [file pone.0137032.s002.docx]

**S1 Table**

| No | Protein Knocked Out | Strain Descriptor | Strain ID | Sources (Depositor) | Description | Blebbing (times/5 min) | n |
| --- | --- | --- | --- | --- | --- | --- | --- |
| 1 | Wild type | AX2 |  | T. Q. P. Uyeda | Wild type | 14.5 ± 7.17 | 60 |
| 2 | GbpC/GbpD  double null mutant | gbpC-/gbpD- | DBS0235996 | stock center (P. van Haastert) | Cyclic GMP binding protein, with RasGEF domain | 42.0 ± 13.8 | 23 |
| 3 | PkgB | pkgB- | DBS0304638 | stock center (P. Devreotes) | SGK family protein kinase | 36.0 ± 22.7 | 20 |
| 4 | IplA | iplA- | DBS0236260 | sock center (R. Kay) | Inositol 1,4,5-trisphosphate receptor-like protein A | 24.8 ± 8.7 | 20 |
| 5 | Pi3k ABCFG quinple null mutant | pikA-/pikB-/pikC-/pikF-/pikG- | DBS0252652 | stock center (Rob Kay) | PI3-kinase | 22.4 ± 11.9 | 22 |
| 6 | PakA | pakA- |  | M. Iijima | P21-activated protein kinase | 20.0 ± 7.4 | 20 |
| 7 | PlaA | plaA- | DBS0306196 | NBRP (H. Kuwayama) | Phospholipase A2 | 4.3 ± 3.0 | 24 |
| 8 | PTEN | pten- |  | M. Iijima | Phosphatase and Tensin homolog, 3-phosphatidylinositol 3-phosphatase | 2.9 ± 3.3 | 20 |
| 9 | PkbA | pkbA- | DBS0236784 | stock center (R. Firtel) | AKT/PKB protein kinase | 1.4±1.6 | 24 |
| 10 | PakB | pakB- | DBS0236709 | stock center (Graham Cote) | P21-activated protein kinase | 0.8 ± 1.8 | 20 |
| 11 | PTEN/PakA double null mutant | pten-/pakA- |  | M. Iijima | PTEN and PakA double knockout | 0.3 ± 0.8 | 22 |
| 12 | PakC | pakC- | DBS0236716 | stock center (R. Firtel) | P21-activated protein kinase | 0.4 ± 0.9 | 26 |
| 13 | RasC/RasG double null mutant | rasC-/rasG- | DBS0236858 | stock center (P. Bolourani) | Ras GTPase | 19.1 ± 10.5 | 24 |
| 14 | PakB/PakC double null mutant | pakB-/pakC- | DBS0236715 | stock center (R. Firtel) | P21-activated protein kinase | 16.4 ± 8.0 | 19 |
| 15 | RIP3 | ripA- | DBS0236900 | stock center (R. Firtel) | Ras-interacting protein | 13.6 ± 8.8 | 27 |
| 16 | Gca/SgcA double null mutant | gca-/sgcA- | DBS0236000 | stock center (P. van Haastert) | GcA: Gunanylyl cyclase, SgcA: Soluble guanylyl cyclase | 10.2 ± 5.0 | 21 |
| 17 | LvsA | lvsA- | DBS0309063 | stock center (P. Cosson) | BEACH domain-containing contractile vacuole protein | 36.4 ± 18.9 | 18 |
| 18 | EpnA | epnA- | DBS0302448 | stock center (T. O'Halloran) | Epsin, which interacts with clathrin-coated pits | 6.8 ± 4.9 | 19 |
| 19 | DymA | dymA- |  | S. Miyagishima | Dynamin A | 6.5 ± 6.6 | 20 |
| 20 | Annexin | nxnA- | DBS0236955 | stock center (A. Noegel) | Calcium-regulated phospholipid- and membrane-binding protein | 11. 9 ± 9.6 | 20 |
| 21 | DlpA | dlpA- |  | S. Miyagishima | Dynamin-like protein A | 11.8 ± 8.1 | 20 |
| 22 | Cortexillin I/II  double null mutant | ctxA-/ctxB- | DBS0235599 | stock center  (G. Gerisch) | Subfamily of proteins with actin-binding sites of the alpha-actinin/spectrin | 56.7 ± 20.3 | 21 |
| 23 | LimB | limB- | DBS0237631 | stock center (K. Weijer) | LIM domain-containing protein. LIM domain controls cytoskeletal rearrangements, including paxillin, | 38.9 ± 26.4 | 11 |
| 24 | Kif12 | kif12- | DBS0236489 | stock center (T. O'Halloran) | MKLP1 subfamily kinesin | 32.2 ± 15.1 | 20 |
| 25 | GefB | gefB- | DBS0236009 | stock center (R. Insall) | Ras guanine nucleotide exchange factor | 32.5 ± 21.3 | 18 |
| 26 | SCAR1 | scrA- | DBS0236435 | stock center (R. Insall) | Adaptor protein that couples Rho GTPases and the Arp2/3 complex to stimulate actin polymerization. | 2.7 ± 4.7 | 21 |
| 27 | Myosin II | mhcA- | S00023 | NBRP  (J. Spudich) | Myosin II heavy chain | 0.4 ± 1.0 | 21 |
| 28 | AbpA/AbpC/Sev triple mutant | abpA-/abpC-/sevA- | DBS0236169 | stock center (G. Gerisch) | Alpha-actinin, 120 kDa gelation factor, and severin | 8.9 ± 9.5 | 21 |
| 29 | VASP | vasP- | DBS0237035 | stock center (R. Firtel) | VASP family protein | 7.7 ± 11.0 | 17 |
